# Selective Inhibition of Coxiella burnetii Replication by the Steroid Hormone Progesterone

**DOI:** 10.1128/IAI.00894-19

**Published:** 2020-11-16

**Authors:** Zachary P. Howard, Anders Omsland

**Affiliations:** a Paul G. Allen School for Global Animal Health, Washington State University, Pullman, Washington, USA; b School of Molecular Biosciences, Washington State University, Pullman, Washington, USA; Yale University School of Medicine

**Keywords:** *Coxiella burnetii*, progesterone, steroid hormone, intracellular replication, axenic, efflux pumps

## Abstract

Coxiella burnetii is a zoonotic bacterial obligate intracellular parasite and the cause of query (Q) fever. During natural infection of female animals, C. burnetii shows tropism for the placenta and is associated with late-term abortion, at which time the pathogen titer in placental tissue can exceed one billion bacteria per gram. During later stages of pregnancy, placental trophoblasts serve as the major source of progesterone, a steroid hormone known to affect the replication of some pathogens. During infection of placenta-derived JEG-3 cells, C. burnetii showed sensitivity to progesterone but not the immediate precursor pregnenolone or estrogen, another major mammalian steroid hormone. Using host cell-free culture, progesterone was determined to have a direct inhibitory effect on C. burnetii replication. Synergy between the inhibitory effect of progesterone and the efflux pump inhibitors verapamil and 1-(1-naphthylmethyl)-piperazine is consistent with a role for efflux pumps in preventing progesterone-mediated inhibition of C. burnetii activity. The sensitivity of C. burnetii to progesterone, but not structurally related molecules, is consistent with the ability of progesterone to influence pathogen replication in progesterone-producing tissues.

## Abstract (precis)

Coxiella burnetii is a zoonotic bacterial obligate intracellular parasite and the cause of query (Q) fever. During natural infection of female animals, C. burnetii shows tropism for the placenta and is associated with late-term abortion, at which time the pathogen titer in placental tissue can exceed one billion bacteria per gram. During later stages of pregnancy, placental trophoblasts serve as the major source of progesterone, a steroid hormone known to affect the replication of some pathogens.

## INTRODUCTION

Coxiella burnetii is a zoonotic, Gram-negative bacterial pathogen and the causative agent of Q fever (1). C. burnetii is an obligate intracellular parasite that invades eukaryotic cells and subsequently replicates within a phagolysosome-derived vacuole referred to as the *Coxiella*-containing vacuole (CCV) (1, 2). C. burnetii is known to colonize the placenta and cause reproductive disorders, such as abortions, in animals (3). In fact, C. burnetii is typically shed into the environment via birth products of domestic ruminants; human infection can occur after inhalation of pathogen-contaminated aerosols or via ingestion of certain animal products (e.g., unpasteurized milk) (1, 3). Interestingly, males are more likely to develop symptomatic Q fever and experience more severe symptoms than females (4, 5). However, the molecular mechanisms implicated in sex-dependent infection phenotypes remain unclear.

Independent lines of evidence point to a role for sex hormones in the observed sex-dependent disease severity of Q fever. 17β-Estradiol, the predominant estrogen hormone, has been shown to confer a protective effect in mice infected with C. burnetii (4). Moreover, progesterone, the primary progestogen hormone regulating mammalian pregnancy, has been shown to affect C. burnetii intracellular replication, suggesting a host-mediated effect on pathogen replication (6). In addition to C. burnetii, Neisseria gonorrhoeae replication in nutrient broth can be inhibited by progesterone (7), and N. gonorrhoeae strains that lack genes for resistance-nodulation division (RND) and multidrug resistance (MDR) efflux pumps are more susceptible to progesterone and less viable in a mouse model of genital tract infection (8). Efflux proteins are responsible for pumping toxins and other compounds out of the bacterial cell. Although direct inhibition by female sex hormones was not definitively determined to be responsible for the reduced bacterial viability in the mouse model of N. gonorrhoeae infection, the results support a role for sex hormones in the reproductive pathogenesis of bacteria. For C. burnetii, bacterial loads have been observed to increase toward parturition (3), when the physiological concentration of progesterone decreases, consistent with a bacterial response to changes in hormone concentrations. Thus, progesterone may be a relevant factor in C. burnetii pathogenesis and pathogen replication in placental tissue.

In this study, we characterize the effects of major mammalian sex hormones on C. burnetii replication. Using host cell-free culture tools to isolate the pathogen from the host cell and the placental (choriocarcinoma) JEG-3 cell line to assess pathogen responses to hormones under intracellular replication, we show that C. burnetii is directly and selectively inhibited by progesterone but not structurally related steroid hormones.

## RESULTS

### Progesterone inhibits replication of C. burnetii.

**FIG 1:**
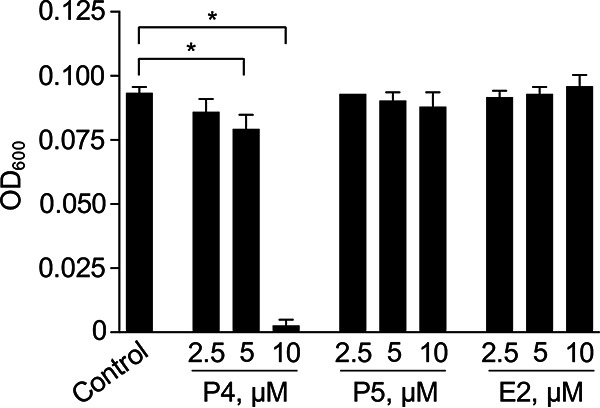
Progesterone inhibits C. burnetii replication in axenic culture. ACCM-1 was inoculated with 1 × 10^6^ C. burnetii per ml and incubated at 37°C for 8 days under concentration gradients of P4, P5, and E2. Culture optical density (OD_600_) was measured on day 8. Depicted data represent the means from three independent experiments, and error bars represent standard errors of the means (SEMs). *, *P* < 0.05 (one-way analysis of variance [ANOVA] and Sidak’s multiple-comparison test).

C. burnetii infects and replicates in mammalian placental tissue and is known to cause spontaneous abortion in ruminants (1, 3). The placenta is a source of the sex hormone progesterone (P4) during pregnancy, and several studies have demonstrated that P4 can directly inhibit bacterial replication. For example, 127 μM P4 has been shown to directly inhibit N. gonorrhoeae replication (7). We hypothesized that C. burnetii tropism for placental tissue may correlate with pathogen sensitivity to female sex hormones. Thus, the effect of 17β-estradiol (E2) and P4 on C. burnetii replication during axenic culture was tested by incubation of C. burnetii in acidified citrate cysteine medium (ACCM-1) containing various concentrations of the hormones (Fig. 1). ACCM-1 (9), a medium not supplemented with methyl-β-cyclodextrin (MβCD), was used. Cyclodextrins are known to sequester cholesterol (10) and thus potentially also cholesterol-derived hormones such as progesterone. In addition to E2 and P4, the P4 precursor pregnenolone (P5) was included to assess the effect of a structurally highly similar molecule. While no inhibitory effect was observed upon incubation with E2, suggesting that estrogens do not have a direct effect on C. burnetii replication (Fig. 1), incubation with 10 μM P4 completely inhibited replication of C. burnetii in ACCM-1 compared to that observed in the untreated control (Fig. 1). Five micromolar P4 resulted in minor but statistically significant inhibition of C. burnetii replication. Interestingly, no significant inhibition was observed upon incubation with 10 μM P5, suggesting that the inhibitory effect is highly structure-specific, as P5 is the immediate precursor of P4 (Fig. 1). To assess whether P4 affects C. burnetii susceptibility to traditional antibiotics, the combined effect of P4 and doxycycline on bacterial growth was measured. Supplementation of ACCM-1 with subinhibitory concentrations of P4 resulted in a moderate increase in C. burnetii sensitivity to doxycycline (see Fig. S1 in the supplemental material).

**FIG 2:**
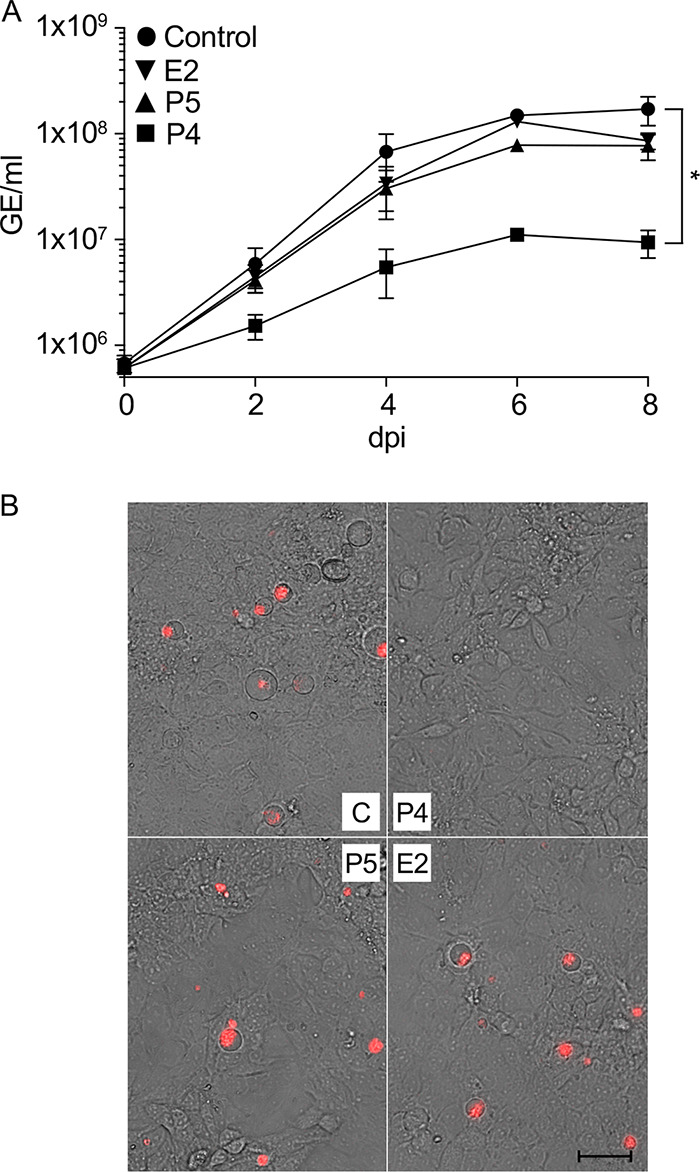
C. burnetii replication in JEG-3 cells is inhibited by progesterone. C. burnetii replication in JEG-3 cells in the presence of exogenous P4, P5, or E2 was evaluated by analysis of GE (A) and fluorescence microscopy (B). JEG-3 cells were infected with C. burnetii and bacterial GE measured every 2 days from 0 to 8 dpi. Data points represent mean GEs from three independent experiments, and error bars represent SEMs. JEG-3 cells infected with C. burnetii expressing mCherry were used to visualize CCV development at 4 dpi. *, *P* < 0.05 (one-way ANOVA with Sidak’s multiple-comparison test, 8 dpi). Scale bar = 50 μm.

To determine if P4 could also inhibit C. burnetii during infection of host cells, we utilized JEG-3 cells, a human choriocarcinoma-derived cell line, as a model for placental tissue. JEG-3 cells were infected with C. burnetii and incubated in cell culture medium supplemented with 10 μM P4, P5, or E2. Similar to the results obtained following incubation in axenic medium, C. burnetii replication was significantly inhibited by P4 during intracellular growth (Fig. 2A). In addition to inhibition of replication, no obvious CCVs were observed in C. burnetii-infected cells cultured in the presence of P4 (Fig. 2B). Importantly, no significant inhibition was observed with P5 or E2, and CCVs appeared normal compared to those observed under control conditions (Fig. 2A and B).

**FIG 3:**
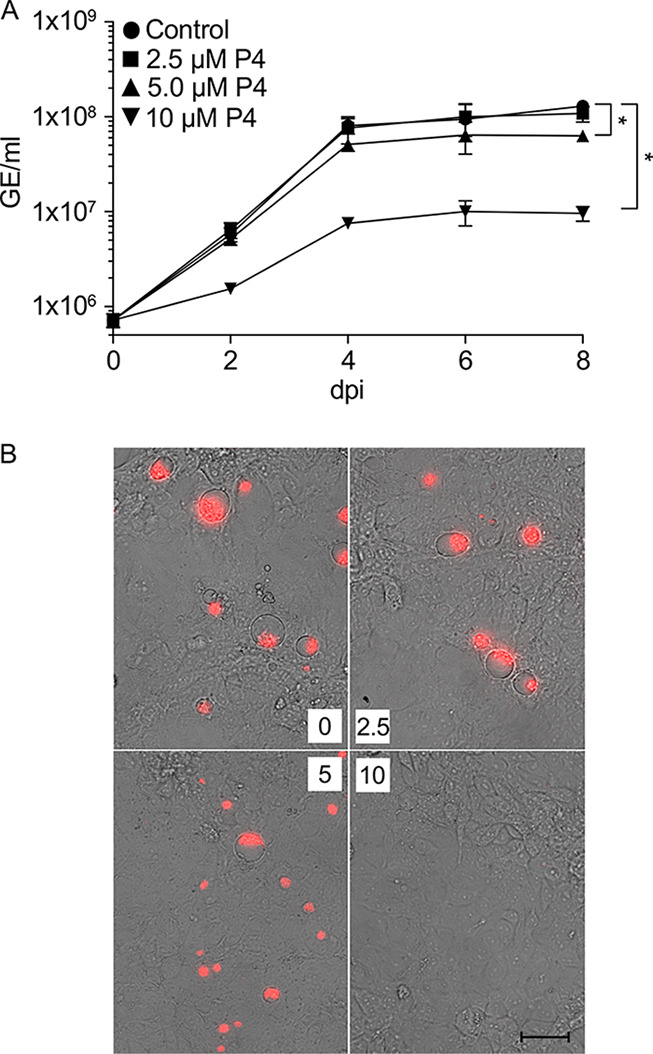
C. burnetii inhibition by progesterone in JEG-3 cells is dose dependent. C. burnetii replication in JEG-3 cells under a concentration gradient of P4 was evaluated by analysis of GE (A) and fluorescence microscopy (B). JEG-3 cells were infected with C. burnetii and bacterial GE measured every 2 days from 0 to 8 dpi. Data points represent mean GEs from three independent experiments, and error bars represent SEMs. JEG-3 cells infected with C. burnetii expressing mCherry were used to visualize CCV development at 4 dpi. *, *P* < 0.05 (one-way ANOVA and Sidak’s multiple-comparison test, 8 dpi). Scale bar = 50 μm.

To determine the minimal concentration of P4 necessary to affect C. burnetii intracellular replication, pathogen-infected JEG-3 cells were incubated in plain medium (control) or in medium supplemented with 2.5, 5, or 10 μM P4 (Fig. 3). Similar to results obtained from experiments conducted under axenic conditions, 10 μM P4 was the apparent minimal concentration required to inhibit both C. burnetii replication and CCV formation in JEG-3 cells (Fig. 3A and B). Overall, these results suggest that P4 is inhibiting C. burnetii replication by acting directly on the pathogen during infection of host cells.

### Progesterone exerts a bacteriostatic effect on C. burnetii.

**FIG 4:**
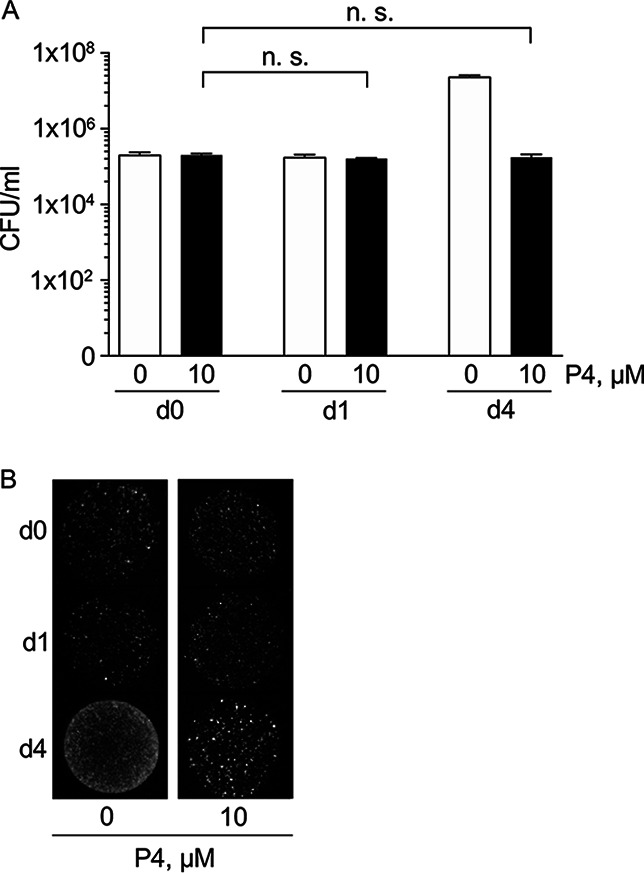
Progesterone does not affect C. burnetii viability in ACCM-1. (A) The number of viable C. burnetii cells following culture in ACCM-1 with or without P4 was determined by CFU analysis. Data points represent mean CFU/ml from three independent experiments, and error bars represent SEMs. (B) Images of representative plates spotted with C. burnetii on days 0, 1, and 4 post progesterone challenge. n.s., not statistically significant.

To determine if the effect of P4 on C. burnetii was bactericidal or bacteriostatic, a CFU assay was performed to determine pathogen viability during culture in ACCM-1 in the absence or presence of 10 μM P4. The number of viable bacteria was not affected during 4 days of incubation in ACCM-1 with 10 μM P4 (Fig. 4A). Representative images of spot-plated culture material further demonstrate that 10 μM P4 does not reduce C. burnetii viability in ACCM-1 (Fig. 4B), suggesting that P4 exerts a bacteriostatic effect on C. burnetii in ACCM-1.

**FIG 5:**
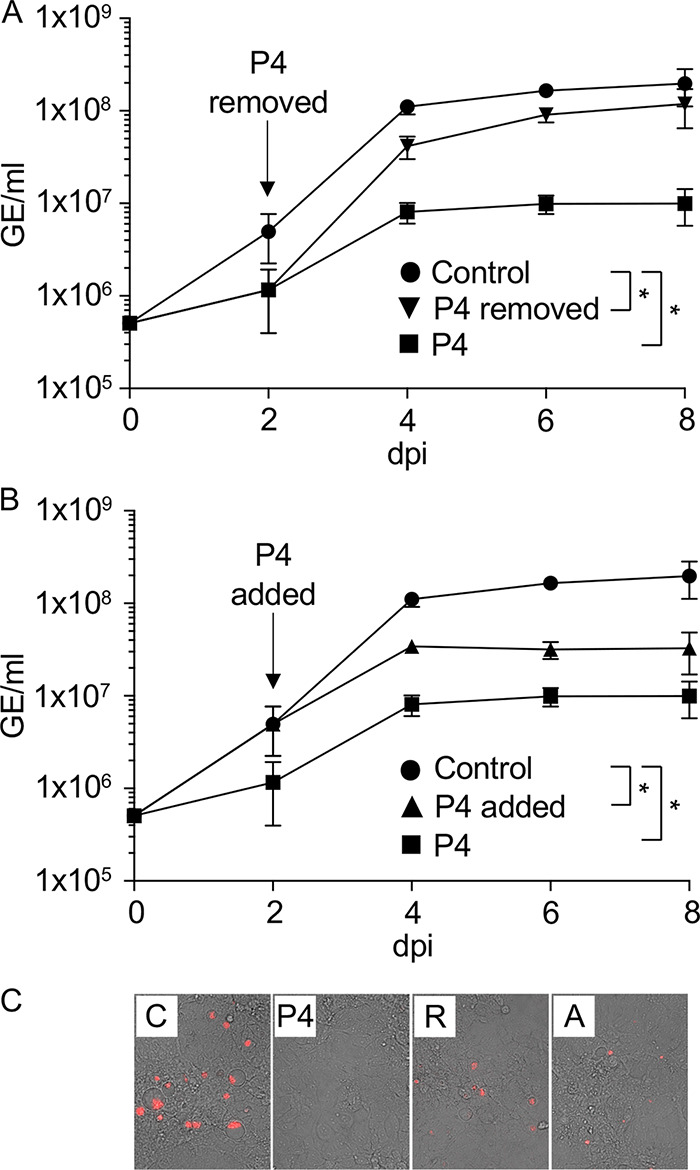
Removing or adding progesterone during C. burnetii infection of JEG-3 cells rescues or inhibits replication, respectively. C. burnetii replication in JEG-3 cells was evaluated by analysis of GE and fluorescence microscopy upon removal (A) or addition (B) of 10 μM P4 at 2 dpi. JEG-3 cells were infected with C. burnetii, and C. burnetii GE was measured every 2 days from 0 to 8 dpi. Data points represent average GEs from three independent experiments, and error bars represent SEMs. (C) JEG-3 cells infected with C. burnetii expressing mCherry were cultured with or without 10 μM P4 until 2 dpi and imaged at 4 dpi, after treatment. *, *P* < 0.05 (one-way ANOVA and Sidak’s multiple-comparison tests, 4 dpi). All conditions were tested as direct comparisons, and the graphs were split to highlight the effect of P4 removal or addition, respectively. C, control; R, removal; A, addition.

In host cells, C. burnetii replicates exclusively within the CCV, a compartment with phagolysosome-like characteristics (11). Thus, inhibition of C. burnetii replication by P4 during infection of host cells could make C. burnetii susceptible to host antimicrobial processes and thus lead to reduced pathogen viability. To test C. burnetii fitness in JEG-3 cells cultured with P4, medium containing P4 was replaced with plain medium at 2 days postinfection (dpi). After replacement of the culture medium, C. burnetii genome equivalents (GE) increased approximately 1 log, and CCVs were visible (Fig. 5A and C). This indicates that P4 has a bacteriostatic effect on C. burnetii during infection of host cells, similar to the observed effect during axenic culture (e.g., Fig. 4). To test if addition of P4 to a progressing infection would inhibit C. burnetii replication, P4 was added 2 dpi, and continuation of replication assessed by analysis of bacterial load by GE (Fig. 5B). When culture medium containing 10 μM P4 was added to infected cells at 2 dpi, replication was significantly inhibited compared to that of the control, and CCVs appeared to condense, indicative of decreased C. burnetii replication and activity (e.g., virulence factor secretion required to maintain the CCV) (Fig. 5B and C). These results further support bacteriostatic inhibition as the effect of P4 on C. burnetii during infection of host cells.

### Efflux inhibitors potentiate the inhibitory effect of P4 on C. burnetii.

**FIG 6:**
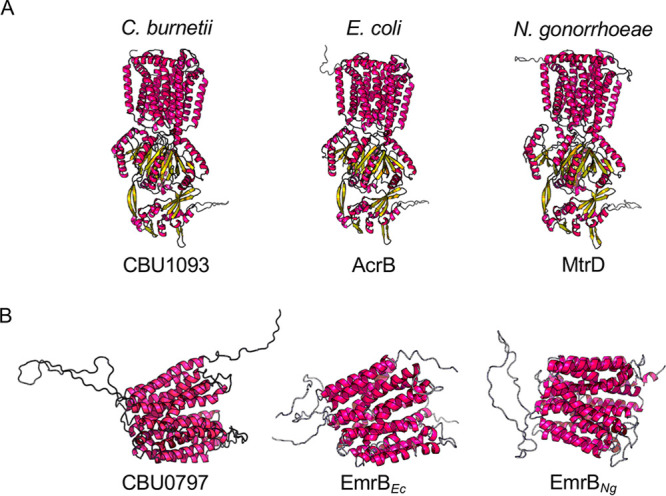
Predicted structures of C. burnetii, E. coli, and N. gonorrhoeae RND- and MFS-type efflux pumps. Progesterone has been shown to be a substrate and competitive inhibitor of RND- and MFS-type efflux systems in N. gonorrhoeae and E. coli. Predicted structures of RND-type (A) and MFS-type (B) efflux pumps in C. burnetii were compared to orthologs in N. gonorrhoeae and E. coli. Predicted protein structures are juxtaposed, and sequence homology displayed as a matrix (Tables 1 and 2).

The results presented in Fig. 1 and 5 demonstrate that C. burnetii replication is inhibited by P4 both under axenic conditions and during infection of JEG-3 cells. However, the mechanism controlling susceptibility to P4-dependent bacteriostasis in C. burnetii was not clear. Bacterial efflux systems are responsible for removing inhibitory compounds from the bacterial cell. For example, N. gonorrhoeae lacking RND efflux genes is more susceptible to inhibition by P4 and was shown to exhibit reduced viability in a mouse model of genital tract infection (8). In Escherichia coli, steroid hormones are substrates for the major RND- and MFS-type multidrug efflux pumps and act as competitive inhibitors for removal of toxic compounds from the bacterial cells (12). Based on the RND-type proteins AcrB and MtrD and MFS-type protein EmrB, CBU0753, CBU0804, and CBU1093 were identified as RND-type pumps, while CBU0797 and CBU1244 were identified as MFS-type efflux pumps in C. burnetii. Amino acid sequence alignment and *in silico* structural prediction (RaptorX [13]) of C. burnetii RND- and MFS-type efflux pumps suggest conservation of structural features with corresponding efflux pump proteins from E. coli and N. gonorrhoeae (Fig. 6A and B; Tables 1 and 2). Despite sequence identities of less than 50%, sequence similarity was as high as 74%. Notably, distantly related efflux pumps in Haemophilus influenzae have similar substrate recognition to efflux pumps from E. coli (14). Thus, the roles of efflux pumps in mediating susceptibility to P4-dependent inhibition observed for N. gonorrhoeae and E. coli are likely to be relevant also for C. burnetii. Interestingly, the C. burnetii genome contains several copies of efflux pump genes (15), including the three genes encoding RND-type efflux proteins, CBU0753, CBU0804, and CBU1093. Additionally, based on searches with CBU1093 and CBU0797 from the C. burnetii reference genome (RSA493), RND- and MFS-type efflux pump genes appear conserved between pathogen isolates. Mutations observed in MFS-type transporters from C. burnetii isolated during the 2007–2010 Q-fever outbreak in the Netherlands (16) further suggest significance for efflux pumps in C. burnetii virulence. Therefore, we hypothesized that C. burnetii efflux pumps may be critical for removal of P4 from the cell and that pharmacological inhibition of efflux pumps may potentiate the effects of P4 on C. burnetii.

**TABLE 1:** Percent identity and similarity between RND-type efflux pumps

Pump	% identity/% similarity				
	AcrB	MtrD	CBU0753	CBU0804	CBU1093
AcrB	100/100^*a*^				
MtrD	49/66	100/100			
CBU0753	31/53	29/50	100/100		
CBU0804	31/50	29/51	46/66	100/100	
CBU1093	30/51	31/54	39/59	39/59	100/100

a 100/100, amino acid percent identity or similarity.

**TABLE 2:** Percent identity and similarity between MFS-type efflux pumps

Pump	% identity/% similarity^*a*^			
	EmrB*_Ec_*	EmrB*_Ng_*	CBU0797	CBU1244
EmrB*_Ec_*	100/100^*b*^			
EmrB*_Ng_*	57/74	100/100		
CBU0797	35/59	35/56	100/100	
CBU1244	24/43	25/43	23/47	100/100

a *Ec*, E. coli; *Ng*, N. gonorrhoeae.

b 100/100, amino acid percent identity or similarity.

**FIG 7:**
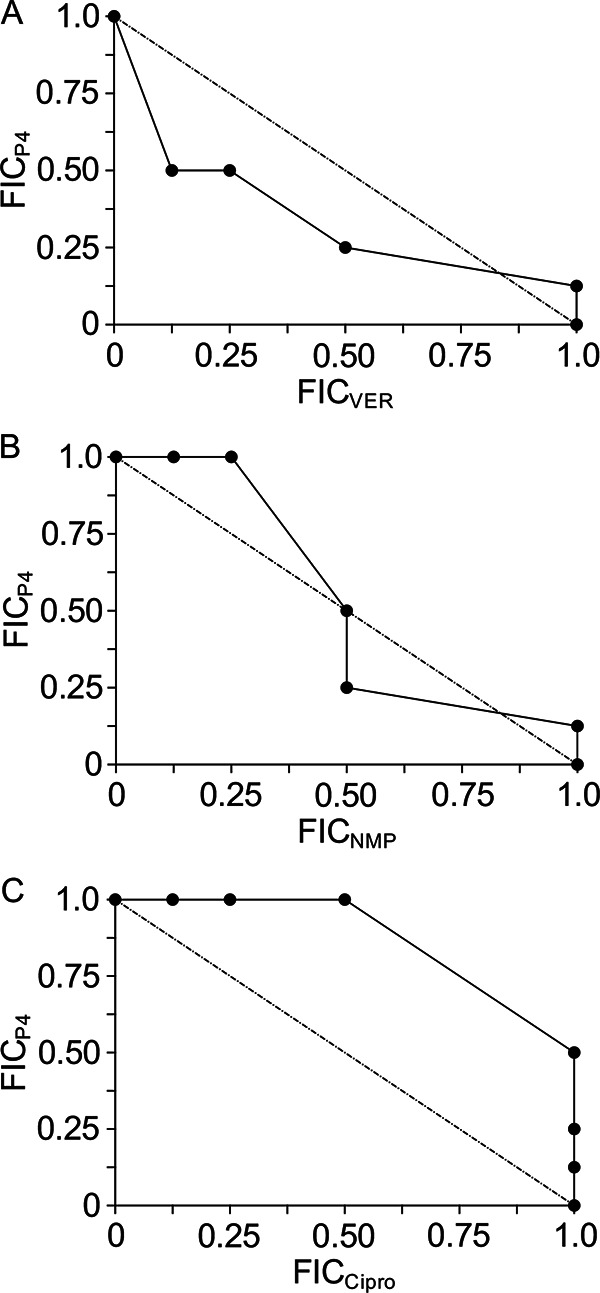
Efflux pump inhibitors potentiate the inhibitory effect of progesterone. Bacteria were cultured in ACCM-1 containing P4, the efflux inhibitors VER and NMP, or a combination of P4 and an efflux inhibitor using a fixed ratio format with the MIC as the highest concentration of each compound. Ciprofloxacin (Cipro), an antibiotic not directly targeting efflux pumps, was used as a negative control for synergistic effects between compounds. Mean OD_600_ values and SEMs from 3 independent experiments were used to calculate fractional inhibitory concentrations and construction of isobolograms for P4/VER (A), P4/NMP (B), and P4/Cipro (C).

To interrogate the role of efflux pumps in mediating the observed P4-dependent inhibition of C. burnetii replication, a checkerboard assay was used to determine if P4 and an efflux pump inhibitor exhibit synergy as inhibitors of C. burnetii growth in ACCM-1 (see Fig. S2). Drug synergy has been defined as a combination with fractional inhibitory concentration (FIC) indices (FICI) of <0.5 (17). Verapamil (VER) is a calcium channel inhibitor that has been characterized as a potent inhibitor of efflux pump activity in bacteria (18). Moreover, VER was demonstrated to potentiate the effect of antituberculosis drugs on Mycobacterium tuberculosis (18). Indeed, upon exposing C. burnetii to VER in combination with P4, a slightly concave isobologram with a minimum FICI of 0.625 was produced (Fig. 7A). The FICI was calculated as the sum of the FICs for P4 (0.5) and VER (0.125) and is represented on the isobologram as the point with the greatest deviation from the line of indifference (Fig. 7A). Additionally, the arylpiperazine 1-(1-naphthylmethyl)-piperazine (NMP), a potent efflux inhibitor in E. coli (19) and Acinetobacter baumannii (20), was tested in combination with P4. Similar to that observed with VER, NMP partially potentiated the inhibitory effect of P4, demonstrated by a partially concave isobologram with a minimum FICI of 0.75 (Fig. 7B). Although the FICI values for P4/VER and P4/NMP are not below the canonical threshold FICI for synergy of 0.5, significant synergistic interactions with FICIs between 0.5 and 0.99 have been demonstrated (21). An analysis of checkerboard assay sensitivity for antifungals demonstrated weaker detection of synergistic interaction at later time points (17) and recommended that the FICI cutoffs be adjusted to <1 for synergy, 1 to 1.25 for indifference, and >1.25 for antagonism. The study also revealed that true synergistic interactions yielded FICIs of less than 1, similar to the results observed here for P4 and VER. Therefore, results presented in Fig. 7 suggest a moderately synergistic interaction between P4 and VER in the context of inhibition of C. burnetii replication.

To validate findings obtained upon treatment with VER and NMP, we used a checkerboard assay to quantify the effect of P4 in combination with the fluoroquinolone antibiotic ciprofloxacin, demonstrated to not be potentiated by inhibition of efflux pumps by carbonyl cyanide *m*-chlorophenyl hydrazine (CCCP) in C. burnetii (22). We first confirmed that the MIC of ciprofloxacin for C. burnetii was 6 μM, as demonstrated previously (22). Indeed, the checkerboard assay revealed a minimum FICI of 1.125 and a convex isobologram, which indicates an indifferent or nonadditive effect of ciprofloxacin on P4-dependent inhibition of C. burnetii replication (Fig. 7C). The lack of a synergistic effect between P4 and ciprofloxacin further supports the evidence for synergy between P4 and VER and thus that P4-dependent inhibition of C. burnetii can be potentiated by efflux pump inhibitors.

### P4 inhibits efflux of ethidium bromide by C. burnetii.

**FIG 8:**
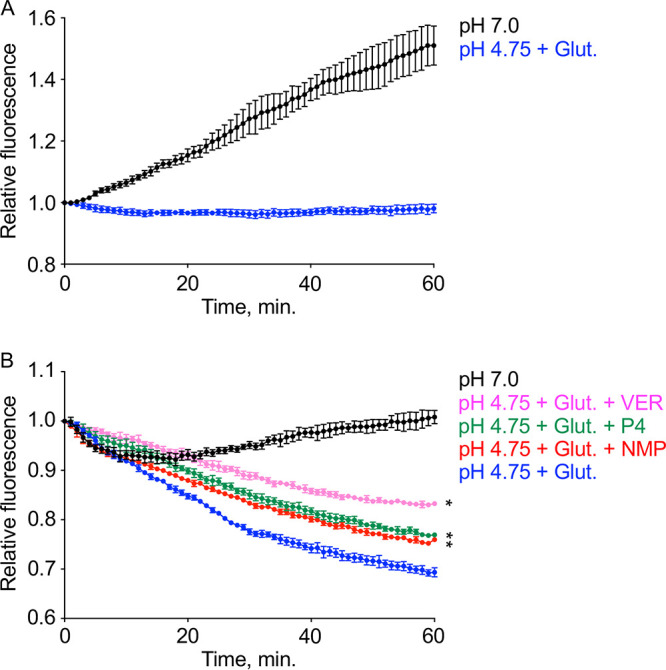
Progesterone inhibits efflux of EtBr. Measurement of bacterial EtBr extrusion was used to test whether P4 inhibits overall bacterial efflux activity. (A) Log-phase C. burnetii was loaded with EtBr for 1 h in ACCM-1 salts (pH 7.0). Incubation at pH 4.75 in the presence of the energy source glutamate prevented loading of cells with EtBr. (B) P4, NMP, and VER added at one-half the respective MICs reduced relative fluorescence compared to that observed under the control condition. Data points represent the means from three independent experiments, and error bars represent SEMs. *, *P* < 0.05 versus pH 4.75+Glut (one-way ANOVA with Sidak’s multiple-comparison test, endpoint).

To confirm the inhibitory effects of P4, VER, and NMP on C. burnetii efflux activity, we measured active efflux of ethidium bromide (EtBr) by log-phase bacteria in the presence or absence of P4, VER, and NMP at one-half the respective MICs, similar to previous studies (23–26). We first determined optimal conditions for loading of C. burnetii with EtBr under energy-deplete conditions (Fig. 8A). Because C. burnetii is unable to generate a proton motive force in buffer at neutral pH (27), we used the buffer base of ACCM-1 (ACCM-1 salts) adjusted to pH 7 for EtBr loading. Indeed, incubation of C. burnetii in ACCM-1 salts at pH 7 resulted in a steady increase in EtBr fluorescence over 1 h at room temperature, indicative of dye loading (Fig. 8A) (pH 7.0). Conversely, when incubated in ACCM-1 salts at the optimal pH for C. burnetii activity (i.e., pH 4.75) with 5 mM glutamate there was no increase in fluorescence (Fig. 8A) (pH 4.75+Glut), indicative of active expulsion of EtBr. Additionally, when P4, NMP, or VER was added at one-half their respective MICs, marginal increases in fluorescence were observed compared to that measured for bacteria incubated at pH 4.75 with 5 mM glutamate, again consistent with inhibition of efflux activity (data not shown).

To confirm the involvement of efflux pumps in affecting C. burnetii susceptibility to P4, we tested if P4 could inhibit the efflux of EtBr. When mid-log-phase C. burnetii cells were loaded with EtBr (Fig. 8A) for 1 h and then reenergized by acidification of the buffer to pH 4.75 in the presence of glutamate, relative fluorescence decreased to 69%, while bacteria incubated under control conditions (pH 7.0) yielded no decrease in relative fluorescence at the final time point (Fig. 8B). The addition of P4 at one-half the MIC resulted in significantly reduced efflux activity as illustrated by retention of the fluorescence signal compared to that of the control, pH 4.75 with glutamate (Fig. 8B). Interestingly, P4 inhibited efflux of EtBr to the same extent as NMP, which indicates P4 is an equally potent inhibitor of efflux as NMP for C. burnetii (Fig. 8B). VER had a greater inhibitory effect on efflux of EtBr than P4 or NMP, which could be due to dissimilar mechanisms of efflux inhibition (Fig. 8B). NMP and P4 likely work via direct binding to efflux pumps, whereas VER works by reducing available energy for secondary transporters such as efflux pumps (12, 28, 29). Importantly, this level of inhibition is consistent with the difference in minimum FICIs observed for VER and NMP, which suggests that VER is a more potent efflux inhibitor than NMP for C. burnetii (Fig. 7).

## DISCUSSION

In this study, we determined the effect of prominent steroid hormones on C. burnetii replication. While E2 and P5 showed minimal effects on C. burnetii growth, P4 resulted in significant inhibition of both replication and CCV development in JEG-3 cells. Using axenic culture to separate C. burnetii from the host cell environment, the results show that P4 primarily acts directly on C. burnetii rather than indirectly via alteration of host physiology. Additionally, P4 inhibition of C. burnetii can be potentiated by the efflux pump inhibitors VER and NMP, which supports the hypotheses that C. burnetii relies on efflux pump activity to overcome P4-dependent bacteriostasis and that P4 is therefore likely a substrate for C. burnetii efflux pumps. In addition to synergy with efflux pump inhibitors, P4 acted as an inhibitor of EtBr efflux, further indicating that P4 is a substrate for C. burnetii efflux pumps. Indeed, the conclusions reached in this study agree with similar studies using N. gonorrhoeae (7) and E. coli (12), which demonstrate that P4 directly inhibits bacterial replication and is a substrate for efflux pumps. In N. gonorrhoeae (7), inhibition of replication by P4 is likely due to membrane binding, which directly inhibits membrane-associated enzymes involved in electron transport. Additional experiments are needed to elucidate the exact mechanism (e.g., role of specific efflux pumps) of P4-dependent inhibition of C. burnetii.

Although the results of this study indicate P4 directly inhibits C. burnetii replication, it is possible that steroid hormones affect C. burnetii replication also via indirect mechanisms. Autophagy is a process used by the host cell to degrade proteins and spent organelles; autophagy is also critical for C. burnetii intracellular replication (30, 31). Studies have demonstrated that high concentrations of P4 can inhibit autophagy in trophoblast cells (32). Therefore, P4-mediated inhibition of autophagy in JEG-3 cells might be partly responsible for the reduced replication observed in our model. However, we were unable to rescue replication by adding rapamycin to induce autophagy or mifepristone to competitively inhibit the progesterone receptor in P4-treated cells (data not shown). P4 has been shown to affect C. burnetii intracellular replication in THP-1 (human monocyte-like) cells with no loss in host cell viability (6). When incubated in ACCM-1, a medium that does not contain the steroid-binding compound methyl-β-cyclodextrin, P4 has a clear direct effect of C. burnetii replication. Together, the presented results are consistent with a direct effect of P4 on C. burnetii intracellular replication, for example, via fluid phase uptake of P4 into the CCV, as shown for fluorescently labeled molecules (33).

The physiological relevance of the results presented herein will require additional scrutiny and ultimately require analysis of C. burnetii replication within placental tissue during different stages of gestation. The concentration of P4 in placental cells colonized by C. burnetii has not been determined. However, placental synthesis of P4 can reach levels as high as 300 mg per day (34), suggesting P4 can reach sufficiently high concentrations in placental tissue to reduce C. burnetii replication. In humans, while the concentrations of P4 in serum during pregnancy have been measured in the range of ∼40 to 170 ng/ml (∼0.1 to 0.5 μM), the concentration of P4 in placental tissue can exceed these levels by 10- to 50-fold (depending on stage of gestation) (35), a concentration range consistent with inhibition of C. burnetii replication. These correlations suggest a model whereby C. burnetii remains latent or replicates at a reduced rate during the majority of pregnancy when placental P4 synthesis remains high and may have increased rates of replication close to parturition, when P4 concentrations decline as required to initiate parturition. Alternatively, C. burnetii loads in placental tissue may be greater for animals with reduced P4 production or animals infected with strains having enhanced efflux capabilities. While we confirmed both basal and P5-dependent synthesis of P4 by JEG-3 cells, accumulation of P4 in culture supernatants did not reach inhibitory (i.e., ∼10 μM) levels within 48 h of culture (data not shown). Regardless, the JEG-3-based model demonstrates P4-dependent inhibition of C. burnetii in the context of intracellular replication. While the ability of C. burnetii to colonize and replicate within a host could be affected by a number of factors, including pathogen isolate (36) and host immune status, our data point to a direct effect of P4 in C. burnetii virulence. Future studies will aim to understand the physiological implications of the described P4-dependent inhibitory effects.

ACCM-1 and not ACCM-2 was used to investigate the inhibitory effects of P4 on C. burnetii replication due to the substitution of MβCD for 1% fetal bovine serum (FBS) in ACCM-1. The MIC of P4 for C. burnetii in ACCM-2, which contains 1 mg/ml (wt/vol) of MβCD was approximately 200 μM (data not shown), 20-fold higher than the MIC in ACCM-1. The antagonistic effect of MβCD is likely due to the ability of this compound to sequester hydrophobic molecules, such as cholesterol (10). We suggest that any study utilizing axenic media to investigate the effects of hydrophobic molecules on C. burnetii should avoid using media containing MβCD.

If efflux pump inhibitors increase C. burnetii susceptibility to steroid hormones, pharmacological treatments which inhibit bacterial efflux pump activity may be exploited as a novel strategy in treating C. burnetii infections. Additionally, enhanced efflux pump expression or activity may be responsible for the observed antibiotic resistance in some C. burnetii isolates (37). Indeed, C. burnetii resistance to doxycycline has been observed, but no mechanism for such resistance has been determined (38). These studies provide rationale to further investigate efflux pump activity in C. burnetii antibiotic tolerance using methods described in this study. While we relied on a pharmacological approach to study C. burnetii efflux pump activity, generation of C. burnetii mutants with defects in genes encoding efflux pumps would be required to identify the significance of specific efflux pumps. However, C. burnetii has several copies of efflux pump genes (15), which makes dissecting the function of these genes in the context of steroid hormone susceptibility via targeted gene inactivation a major challenge in this organism.

### Perspective.

While C. burnetii has a wide host range and can infect various tissues in both male and female animals, the natural history of C. burnetii reflects a principal association with female reproductive tissue and secretions, the placenta and milk (1). C. burnetii infection of ovariectomized mice revealed higher bacterial loads in the livers and spleens of these animals, reaching levels comparable to those in male animals (4). Moreover, C. burnetii infection is associated with more pronounced symptoms in men than in woman (1, 5). Together with the ability of C. burnetii to infect animals for the duration of the host’s life, such association is consistent with persistent maintenance of C. burnetii in animal populations, thus enhancing the epidemiological footprint. Our findings are consistent with a model whereby C. burnetii has evolved tropism for placental tissue in part because P4 production by the placenta retards pathogen replication, thus maintaining pregnancy and the viability of the infected tissue until the placenta can hold the maximal bacterial load possible. It is tempting to speculate that P4 levels in placental tissue during pregnancy reduce C. burnetii replication during gestation, thus preventing preterm abortion. Deposition of placental tissue into the environment late in pregnancy would allow the maximal number of bacterial cells to be shed, thus enhancing the likelihood of C. burnetii transmission to a new host.

## MATERIALS AND METHODS

### Bacteria.

C. burnetii Nine Mile phase II (NMII) clone 4 (RSA439) was used in this study. C. burnetii NMII replicates with similar kinetics in host cell-free medium (39) and in human macrophages as the virulent Nine Mile phase I strain (RSA493) (11).

### Axenic analyses of hormone-dependent inhibition of C. burnetii replication.

Axenic culture of C. burnetii was performed using acidified citrate cysteine medium (ACCM-1) (9) (in the absence of the steroid-binding compound methyl-β-cyclodextrin) at 37°C under microaerobic conditions (5% CO_2_ and 5% O_2_). All cultures were incubated in T-25 cell culture flasks unless otherwise specified, and growth quantified by measuring culture optical density at 600 nm (OD_600_) using a Beckman DU530 spectrophotometer (Beckman Coulter, Indianapolis, IN). Progesterone (P4), 5-pregnen-3β-ol-20-one (pregnenolone [P5]), or 17β-estradiol (E2) (Sigma-Aldrich, St. Louis, MO) were dissolved in dimethyl sulfoxide (DMSO) (Sigma-Aldrich, St. Louis, MO) and diluted in the culture medium to specified concentrations. The concentration of DMSO was normalized across all conditions to control for any effects of the solvent on bacterial replication. Analyses of the effect of hormones on pathogen replication were conducted with the concentration of DMSO below inhibitory concentrations. Bacterial viability was measured using a CFU assay. Ten-microliter samples were spotted in triplicates on ACCM-2 plus tryptophan (0.5 mM) as described previously (40). Samples were stored at −80°C in ACCM-2 plus 10% DMSO until analysis.

### Analyses of hormone-dependent inhibition of C. burnetii during intracellular replication.

JEG-3 cells (HTB-36; ATCC), which naturally produce and respond to P4, were used to model C. burnetii infection of placental tissue *in vitro*. JEG-3 cells were maintained in RPMI 1640 medium without l-glutamine (Corning Cellgro; Corning Inc., Corning, NY) supplemented with GlutaMAX (Gibco BioSciences, Dublin, Ireland) and 10% (vol/vol) heat-inactivated serum complex (hiFetalPlex; Gemini Bio-Products, Sacramento, CA) at 37°C and 5% CO_2_. During infection, the level of serum complex was reduced to 2%. Cells were first seeded at a density of 10^5^ cells per well in 12-well cell culture plates for quantitative analysis or 6-well plates for imaging. Cells were then infected at a multiplicity of infection (MOI) of 5 in 1 ml (12-well plates) or 2 ml (6-well plates) plain RPMI medium supplemented with GlutaMAX by centrifuging plates at 400 × *g* for 30 min at room temperature. C. burnetii NMII pJB-CAT-P1169-mCherry was used in some experiments to facilitate microscopy. After infection, cells were washed twice with phosphate-buffered saline (PBS) containing MgCl_2_ and CaCl_2_ (pH 7.2) before incubation in culture medium containing P4, P5, E2, or DMSO (vehicle control) at specified concentrations. Cells were incubated at 37°C and 5% CO_2_, and culture media were replaced every 2 days during the course of infection. Four samples were collected on day 0 to quantify initial infection, and duplicate samples for each condition were collected every 2 days postinfection in 1 ml total volume of PBS and transferred to a 1.5-ml gasketed tube containing 0.1-mm zirconia beads (Bio Spec Products, Bartlesville, OK). Samples were first heated at 95°C for 5 min prior to mechanical homogenization (FastPrep-24; MP Biomedicals) at 5 m/s for 20 s, three times. C. burnetii genome equivalents (GE) were measured by quantitative PCR (qPCR) using iTaq Universal SYBR green Supermix (Bio-Rad Laboratories, Hercules, CA) and a CFX96 real-time PCR detection system (Bio-Rad Laboratories, Hercules, CA). Primers for the single-copy C. burnetii gene *dotA* were used to quantify C. burnetii genomes, as described previously (41).

### MICs and checkerboard assays.

The MICs of P4, the fluoroquinolone antibiotic ciprofloxacin, and the efflux inhibitors verapamil (VER) and 1-(1-naphthylmethyl)-piperazine (NMP) (Sigma-Aldrich, St. Louis, MO) were determined by culturing C. burnetii for 8 days in 12-well plates containing 1 ml ACCM-1 supplemented with each compound over a concentration gradient. P4 was dissolved in DMSO, NMP was dissolved in 1 M HCl, VER was dissolved directly in ACCM-1 at the maximum concentration tested, and ciprofloxacin was dissolved in sterile ultrapure water (18.2 MΩ, Milli-Q integral water purification systems; EMD Millipore). The MIC was defined as the concentration at which the OD_600_ was <10% of the untreated control after 8 days of culture (17). Percent growth was calculated by dividing the optical density for the test culture (culture exposed to combination of P4 and drug) by that of the untreated control and multiplying by 100 ([OD_test_/OD_untreated_] × 100). MICs were calculated using average OD_600_ values from at least 3 independent experiments.

Checkerboard assays were used to determine the MIC of P4 in combination with NMP, VER, or ciprofloxacin. Briefly, four 2-fold dilutions starting from the MIC of P4, NMP, VER, or ciprofloxacin were made alone or in combination using ACCM-1, and 1 ml was added in duplicate to 12-well plates. Cultures were inoculated with 10^6^ C. burnetii per ml and incubated for 8 days along with untreated and mock-infection controls. OD_600_ was measured for each combination, and MIC_test_ was defined as stated previously. The fractional inhibitory concentration (FIC) and the FIC index (FICI) were calculated as described previously (17). The FIC was calculated by dividing the MIC of the steroid or drug in combination by the MIC of the steroid or drug alone (FIC_P4_ = MIC_P4+drug_/MIC_P4_ or FIC_drug_ = MIC_drug+P4_/MIC_drug_). The FICI was calculated as the sum of the FICs (FIC_P4_ + FIC_drug_) for both drug and P4 at the no-growth boundary in the checkerboard assay. Isobolograms were constructed by plotting the FIC for all combinations tested of P4 and the drug that yielded no growth.

### Real-time ethidium bromide fluorescence assay for measuring C. burnetii efflux activity.

To measure the efflux activity of C. burnetii, mid-log-phase bacteria were loaded with ethidium bromide (EtBr) (Fisher Scientific, Pittsburg, PA) under energy-deplete conditions before being reenergized and measuring change in fluorescence over time, similar to previous studies (23–26). First, C. burnetii was cultured for 3 days in ACCM-1 plus tryptophan (0.5 mM) to yield a greater number of bacteria for the assay and then pelleted by centrifugation at 15,000 × *g* in a TOMY MX-370 centrifuge (Amuza, Inc., San Diego, CA) at room temperature for 20 min. The pellet was then suspended in ACCM-1 salts (pH 7.0), and the OD_600_ was normalized to 0.5. To measure EtBr loading of cells, 5 aliquots of the bacterial suspension were transferred to 1.5-ml microcentrifuge tubes and centrifuged at 15,000 × *g* for 5 min at room temperature. The pellets were then resuspended to yield a final OD_600_ of 0.25 in ACCM salts (pH 7.0 or pH 4.75) supplemented with 5 mM glutamate with or without P4, NMP, or VER at one-half the respective MIC, containing 0.5 μg/ml EtBr. Two hundred microliters of each sample was transferred to individual wells in a black 96-well plate (Corning Inc., Corning, NY). The fluorescence intensity was measured every minute for 1 h at 37°C in a Spark multimode microplate reader (Tecan, Switzerland) using excitation and emission wavelengths of 480 nm and 630 nm, respectively.

Dye loading was performed using ACCM-1 salts (pH 7.0) to promote the greatest dye uptake. After dye loading, 100-μl aliquots were added to wells of a black 96-well plate (Corning Inc., Corning, NY), and 100 μl of ACCM-1 salts (pH 3.4) supplemented with 10 mM glutamate with or without P4, NMP, and VER at the respective MICs were added to one well each. The final pH was 4.75, which is optimal for C. burnetii replication, and the final concentration of glutamate was 5 mM. The final concentrations of P4, NMP, and VER were one-half the respective MICs. Relative fluorescence was calculated by dividing the fluorescence signal at each time point by the signal obtained at *t* = 0.

### Sequence alignment and prediction of efflux pump structure.

Putative efflux pump genes in C. burnetii were identified using InterPro (42) by searching for predicted proteins within the acriflavine resistance protein (IPR001036) and drug resistance transporter EmrB-like (IPR004638) families and by using the BLASTP suite available from the U.S. National Library of Medicine, National Center for Biotechnology Information against relevant orthologs from E. coli or N. gonorrhoeae. Sequence alignments to determine percent identity and similarity were performed using Clustal Omega (43), EMBL-EBI, and BLASTP. Protein structure was predicted using RaptorX (13). Sequences with coverage of <80% were not considered in the analysis.

### Statistical analysis.

Data were plotted and analyzed using Prism (GraphPad Software, CA).

## Supplementary Material

Supplemental file 1
