# Supplementary material for: Selective Inhibition of Coxiella burnetii Replication by the Steroid Hormone Progesterone
Source: Infect Immun. 2020 Nov 16;88(12):e00894-19. doi: 10.1128/IAI.00894-19 (PMC7671902; doi:10.1128/IAI.00894-19)
Supplement: Supplemental file 1 [file IAI.00894-19-s0001.pdf]

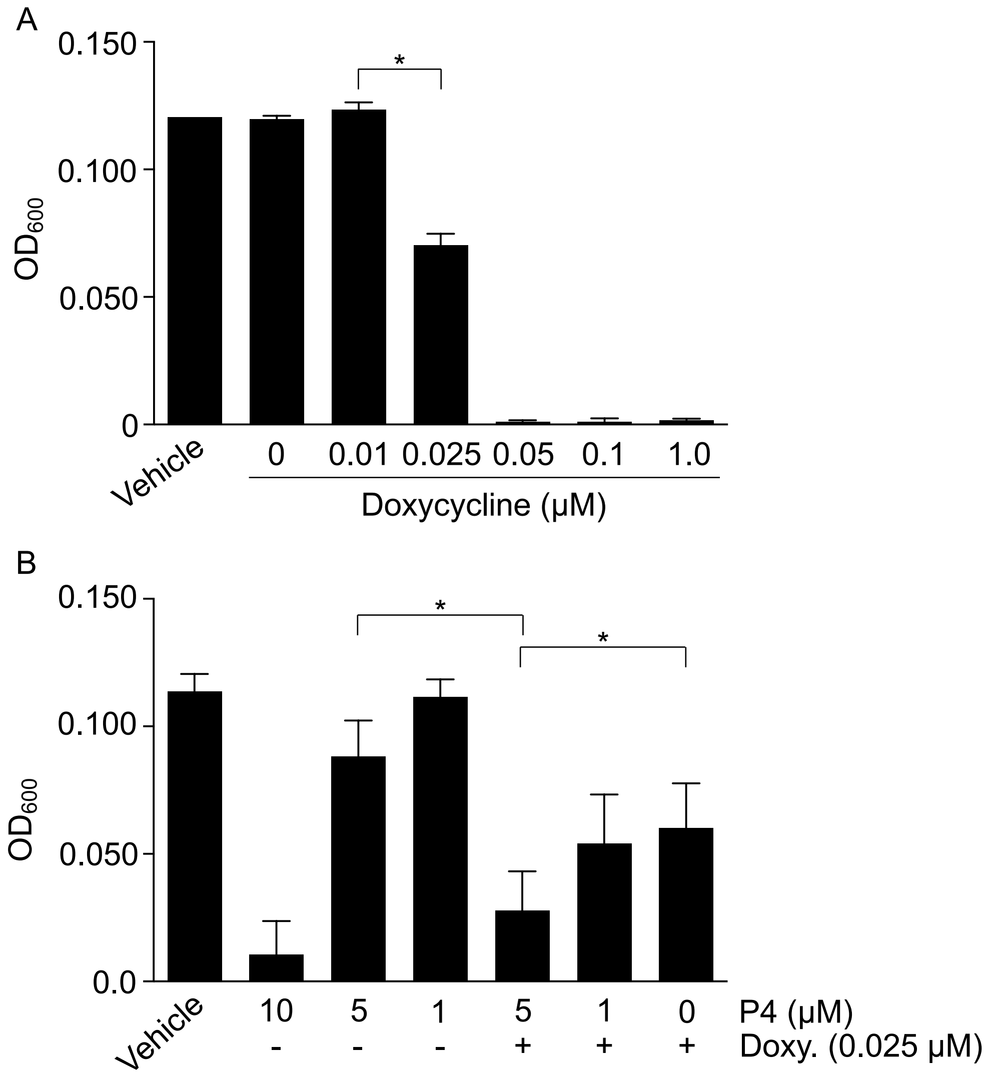

Figure S1. Effect of Progesterone on *C. burnetii* susceptibility to doxycycline. The susceptibility of *C. burnetii* to doxycycline in ACCM-1 was determined by (**A**) analysis of culture optical density (OD_{600}) after 8d of culture. The IC50 for *C. burnetii* susceptibility to doxycycline was 0.025 µM. To test if sub-inhibitory concentrations of P4 potentiates the inhibitory effect of doxycycline *C. burnetii* was (**B**) incubated in ACCM-1 containing P4 alone or in combination with doxycycline. 10 µM P4 was included as a control for susceptibility to the hormone. OD_{600} was measured on day 8. Data points represent the mean of three independent experiments and error bars represent SD. Asterisks denote statistical significance, $P < 0.05$ (One-way ANOVA with Sidak's multiple comparisons test).

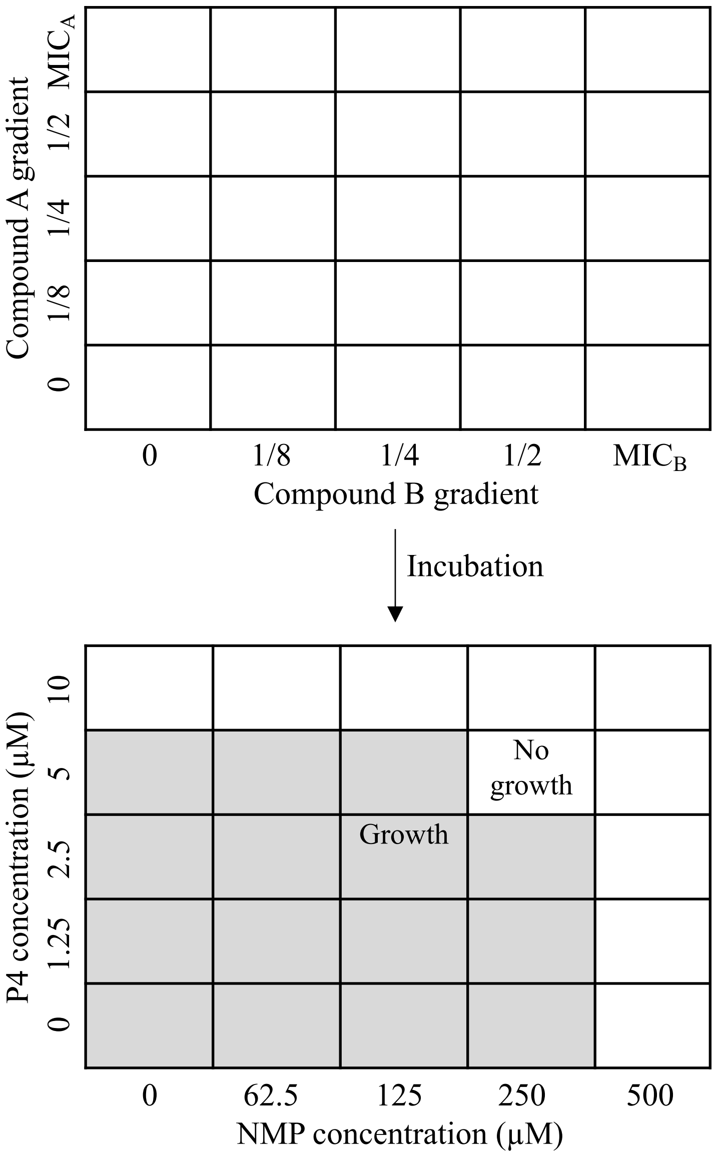

Figure S2. **Schematic of fixed ratio checkerboard assay**. The MICs of compounds A and B were determined, then added to multi-well plates by 2-fold serial dilution. The FICs were calculated based on drug combinations that produced at least 90% inhibition of growth as measured by culture optical density.
